# Differential spatiotemporal gait effects with frequency and dopaminergic modulation in STN-DBS

**DOI:** 10.3389/fnagi.2023.1206533

**Published:** 2023-09-29

**Authors:** Ritesh A. Ramdhani, Jeremy Watts, Myriam Kline, Toni Fitzpatrick, Martin Niethammer, Anahita Khojandi

**Affiliations:** ^1^Department of Neurology, Zucker School of Medicine at Hofstra/Northwell, Hempstead, NY, United States; ^2^Department of Industrial and Systems Engineering, University of Tennessee, Knoxville, TN, United States; ^3^Center for Neurosciences, Feinstein Institutes for Medical Research at Northwell Health, Manhasset, NY, United States

**Keywords:** subthalamic deep brain stimulation, low-frequency, Parkinson's disease, gait kinematics, sensors

## Abstract

**Objective:**

The spatiotemporal gait changes in advanced Parkinson's disease (PD) remain a treatment challenge and have variable responses to L-dopa and subthalamic deep brain stimulation (STN-DBS). The purpose of this study was to determine whether low-frequency STN-DBS (LFS; 60 Hz) elicits a differential response to high-frequency STN-DBS (HFS; 180 Hz) in spatiotemporal gait kinematics.

**Methods:**

Advanced PD subjects with chronic STN-DBS were evaluated in both the OFF and ON medication states with LFS and HFS stimulation. Randomization of electrode contact pairs and frequency conditions was conducted. Instrumented Stand and Walk assessments were carried out for every stimulation/medication condition. LM-ANOVA was employed for analysis.

**Results:**

Twenty-two PD subjects participated in the study, with a mean age (SD) of 63.9 years. Significant interactions between frequency (both LFS and HFS) and electrode contact pairs (particularly ventrally located contacts) were observed for both spatial (foot elevation, toe-off angle, stride length) and temporal (foot speed, stance, single limb support (SLS) and foot swing) gait parameters. A synergistic effect was also demonstrated with L-dopa and both HFS and LFS for right SLS, left stance, left foot swing, right toe-off angle, and left arm range of motion. HFS produced significant improvement in trunk and lumbar range of motion compared to LFS.

**Conclusion:**

The study provides evidence of synergism of L-dopa and STN-DBS on lower limb spatial and temporal measures in advanced PD. HFS and LFS STN-DBS produced equivalent effects among all other tested lower limb gait features. HFS produced significant trunk and lumbar kinematic improvements.

## Introduction

Advanced Parkinson's disease (PD) produces characteristic gait changes that include reduced stride length (Morris et al., 1996, 1998), decreased velocity (Galna et al., 2015), shuffling, multistep turning, and freezing of gait (Okuma, 2014). While high-frequency deep brain stimulation (HFS) (130–185 Hz) of the subthalamic nucleus (STN-DBS) controls levodopa-induced motor complications (i.e., wearing offs, dyskinesia), PD gait tends to worsen over 5–10 years after STN-DBS (George et al., 2010; Castrioto et al., 2011; Fasano et al., 2012; Sidiropoulos et al., 2013; Vercruysse et al., 2014; De Oliveira et al., 2019). L-Dopa has been shown to improve stride length, gait velocity, swing velocity, as well as lower leg and arm swing range of motion (Blin et al., 1991; Rochester et al., 2011; Curtze et al., 2015); however, temporal aspects of gait such as cadence, double limb support, and swing duration are less responsive (Blin et al., 1991; O'Sullivan et al., 1998; Curtze et al., 2015). STN-DBS produces similar gait responses as L-dopa, including improved velocity and stride length, while increasing the amplitude of trunk torsion and flexion along with arm and leg movements (Faist et al., 2001; Ferrarin et al., 2002; Rizzone et al., 2002). Several studies suggest that the combination of L-dopa and STN-DBS engenders synergistic effects on these gait parameters along with reducing gait variability—a clinical marker associated with increased fall risk among PD individuals (Faist et al., 2001; Hausdorff, 2009).

It has been the prevailing notion that high-frequency stimulation is the driving force of this clinical benefit. In fact, there are reports that high-frequency DBS in some instances worsens gait (van Nuenen et al., 2008; Vercruysse et al., 2014). Studies have demonstrated the efficacy of low-frequency stimulation ranging from 60 to 80 Hz of the STN in treating gait disorder and/or freezing of gait (Moreau et al., 2008; Brozova et al., 2009; Ricchi et al., 2012; Sidiropoulos et al., 2013; Ramdhani et al., 2015; Sidiropoulos, 2015; Xie et al., 2015). The sustainability of this benefit remains variable, and little is known as to which patient will show a clinical response as well as its interaction with dopaminergic medication. There is growing evidence that stimulation influences motor circuitry via the modulation of neuronal oscillations (Wingeier et al., 2006; Bronte-Stewart et al., 2009). With respect to 60 Hz DBS, it appears to differentially impact neuronal oscillations in the basal ganglia (Blumenfeld et al., 2015, 2017), underscoring the idea that the frequency modulation of various motor networks and symptoms may exist (Silberstein et al., 2005; Santaniello et al., 2015; Oswal et al., 2016) and that a *one “frequency” size fits* all approach may not be most clinically sound in an advancing disease state.

Therefore, the purpose of this study was to determine whether 60 Hz STN-DBS elicits a differential response in spatiotemporal gait kinematics compared with high-frequency stimulation and to assess the influence that levodopa and stimulation location, in terms of electrode contact activation, have on these features.

## Methods

### Subjects

All subjects had idiopathic PD with bilateral STN-DBS (>3 months) and an underlying gait disorder defined as a score of either 2 or 3 on the gait subscore of the MDS-UPDRS III in the levodopa OFF state. Subjects were excluded from the study if they had cognitive deficits that limited compliance with the study protocol or if vestibular or musculoskeletal problems affecting gait were present. All data collection and analysis were approved by the Institutional Review Boards of the Feinstein Medical Research Institutes/Northwell Health and the University of Tennessee at Knoxville. All subjects provided written informed consent.

### Gait instrumentation

For the gait analysis, the full body opal sensor system (a total of six inertial sensors) from the Mobility Lab System (APDM, Portland, OR) was used. It includes expansive analytical software that measures outcomes from watch size sensors that are tethered to various body regions by Velcro bands. The sensor data are wirelessly streamed to a laptop where Mobility Lab software generated the gait and balance metrics.

### Procedures

Participants were evaluated in the following six conditions: OFF DBS/OFF MED, OFF DBS/ON MED, HFS DBS/OFF MED, HFS DBS/ON MED, LFS DBS/OFF MED, and LFS DBS/ON MED.

#### DBS reprogramming

Each electrode contact was reprogrammed by the principal investigator in the OFF medication state under high-frequency stimulation (HFS; 180 Hz). For each of the four contacts on both electrodes, the amplitude was slowly increased by 0.1 mA increments until sustained sensory or motor side effects were produced in HFS. An amplitude of approximately 10% below the side effect threshold was used for both electrodes along with a standard pulse width of 60 μs. Low-frequency stimulation (LFS; 60 Hz) amplitude was determined for each contact by using the HFS amplitude and the total electrical energy delivered (TEED) (Koss et al., 2005):


$$\begin{array}{l} TEED\;=\;\frac{current\;x\;frequency\;x\;pulse\;width}{impedance}. \end{array}$$

Any LFS amplitudes that produced side effects were lowered by 0.1 mA until the symptom(s) disappeared.

#### Study phases

##### Stage I

The primary goal of Stage I was to establish a baseline gait kinematic profile without the effects of stimulation using objective sensor measurements.

Each subject was evaluated during an in-laboratory session under two conditions: the practically defined OFF state (OFF DBS/OFF MED) and the L-Dopa ON State (OFF DBS/ON MED). The medication OFF state was achieved following an overnight withdrawal of all dopaminergic medications, while the DBS OFF state consisted of a 50-min “wash-out” period prior to evaluation. To ensure the transition to the ON medication state, participants were administered 1.5 times their usual L-Dopa dose (up to 300 mg) using carbidopa/levodopa 25–100 tablet(s). Participants were allotted up to 1 h to transition to the ON MED state or sooner if they felt that the dose had taken clinical effect.

Total body opal sensors were affixed to the wrist, feet, sternum, and lumbar spine (L5 level) for the gait assessment. A trained research coordinator instructed each participant to conduct the Stand and Walk (SAW) test which consisted of a 30 s standing period, followed by a 7 m walk at a comfortable speed, a 180-degree turn, and a return 7 m walk. Both of the OFF DBS (OFF and ON) MED conditions consisted of one gait trial with two SAW tests conducted for each trial to capture a sufficient number of gait cycles.

Cognitive function in the ON state was assessed via the Mini-Mental Status Exam (MMSE). A movement disorders specialist (RR, MN) assessed axial symptoms by the MDS-UPDRS III (Goetz et al., 2008) subscores [3.1, 3.3(a-e), 3.9, 3.11, 3.12, 3.13] for facial masking, speech, limb rigidity, freezing of gait, and posture and postural instability during each condition.

##### Stage II

The primary goal of Stage II was to investigate the impact of stimulation frequencies (low and high) and L-Dopa on gait kinematic domains. Participants were evaluated in two conditions: (1) ON DBS/OFF MED and (2) ON DBS/ON MED.

The opal body sensors (APDM, Opal, Portland, and OR) were affixed to each subject's wrists, ankles, sternum, and lumbar spine, and the SAW assessments were carried out on low- and high-frequency stimulation for the four contact pairs in monopolar settings with the cathode on the contact and anode on the case [e.g., (*Right*) 1- C +/ (*Left*) 1- C+]. All subjects had one of the following DBS systems: St. Jude Medical Infinity DBS System (Abbott Laboratories, Chicago, IL, USA) or Medtronic (Medtronic, Dublin, Ireland). Labeling of the electrode contacts was standardized among the DBS systems with contact 1 indicating the most ventral contact level and contact 4 referring to the most dorsal contact level.

The gait measurements and transition to the ON MED state were the same as in Stage I. Following each stimulation parameter change, there was a 10-min accommodation period before the study assessments were conducted. The participants were blinded to each stimulation change and the contact pair-stimulation mode (HFS/LFS) was randomized, with each subject completing four HFS and four LFS trials (total eight trials) per each medication condition (OFF/ON MED) in the ON DBS state. The medication conditions were not randomized.

Walking aids were utilized in both stages if participants could not safely complete the walk. The presence of L-Dopa-induced dyskinesia was determined by the principal investigator for each of the trials.

### Gait kinematic measures

The measurements of interest captured during the instrumented walk included (R/L: Right/Left) the following:

- Gait—gait cycle duration (seconds, R/L), foot speed (m/s, R/L), stance (%GCT, R/L), swing (%GCT, R/L), heel strike angle (degrees, R/L), toe-off angle (degrees, R/L), stride length (cm, R/L), cadence (steps/min, R/L), step duration (seconds, R/L), elevation at midswing (cm, R/L) (foot clearance, R/L), single limb support (SLS) (%GCT, R/L), arm swing velocity (deg/s, R/L), and range of motion (degrees, R/L).
- Circumduction—turn angle (degrees), turn duration (seconds), and turn velocity (deg/s)
- Lumbar—sagittal range of motion (SRM, degrees), transverse range of motion (TRM, degrees), coronal range of motion (CRM, degrees)
- Trunk—sagittal range of motion (SRM, degrees), transverse range of motion (TRM, degrees), coronal range of motion (CRM, degrees).

### Statistical analysis

Descriptive statistics such as frequencies and percentages were calculated for the categorical variables (e.g., gender, ethnicity, and hemibody first affected); medians, means, and standard deviations were calculated for the numerical variables (i.e., age, disease duration, and LEDD) The Wilcoxon signed-rank test was used to determine differences between the left- and right-side gait parameters (*P* < 0.05 was considered statistically significant). A linear mixed-effects analysis of variance (LM-ANOVA) model was used to determine the effect of L-Dopa, frequency, the interaction of levodopa and frequency, and the interaction of frequency and contact pairs on gait parameters. The fixed effects were L-Dopa condition (ON vs. OFF), stimulation frequency (LFS; 60 Hz vs. HFS; 180 Hz), contact pairs [1-(R) / 1-(L); 2-(R) / 2-(L); 3-(R) / 3-(L); 4-(R) / 4-(L)], and assistive device (presence vs. absence), and the patient effect was considered random. To account for the correlation in repeated measurements (i.e., multiple trials) as well as possible changes in variances/standard deviations over time, an unstructured covariance pattern was assumed. Multiple pairwise comparisons were made using Tukey's procedure, with the overall alpha level set at 0.05. All analyses were conducted using SAS, release 3.8 Enterprise Edition. Copyright 2012–2018, SAS Institute Inc., Cary, NC.

## Results

### Subject characteristics

Twenty-two subjects participated in the study. The mean (SD) age was 63.6 (9) years, 16M/6F with a mean (SD) disease duration of 14.4 (7.8) years. All subjects were on L-dopa treatment for PD with an average LEDD (SD) of 602 (319). The mean (SD) right and left STN stimulation duration was 48.8 (34.9) and 47.6 (35.3) months, respectively. Twelve subjects had the St. Jude Infinity DBS System (Abbott Laboratories, Chicago, IL), and 10 subjects had the Medtronic DBS System (Medtronic, Dublin, Ireland). Segmented leads were utilized in 12 of the 22 subjects. Table 1 summarizes the baseline clinical characteristics: PD phenotypes (N): tremor dominant was 4 (18%), postural instability and gait disorder was 6 (27%), and intermediate was 12 (54%). The mean (SD) baseline (OFF DBS/OFF MED) axial subscore was 11.14 (4.99), and the mean (SD) gait subscore was 2.4 (0.9). The mean (SD) MMSE for the cohort was 26 (2.55). An assistive device was used by 10 subjects and in 112 of a total of 723 SAW trials.

**Table 1:** Subject demographics and clinical characteristics (means and standard deviations).

**Age (y)**	**63.59 (9.12)**
Sex (males/females)	16/6
Disease duration (y)	14.4 (7.8)
PD phenotype	
Tremor dominant (*N*)	4
PIGD (*N*)	6
Intermediate (*N*)	12
Hemibody first affected	
Left (*N*)	10
Right (*N*)	12
DBS duration (mos.)	
Right STN	48.77 (34.87)
Left STN	47.64 (35.33)
LEDD	602.09 (319.04)
Height (in)	67.44 (2.39)
Weight (kg)	81.97 (17.35)
MDS-UPDRS III Axial^*^ Subscore OFF DBS/OFF MED	11.14 (4.99)
MDS-UPDRS III Axial Subscore OFF DBS/ON MED	8 (7.27)
MDS-UPDRS III Gait Subscore	2.4 (0.9)
MMSE	26.73 (2.55)

^*^Sum of subscores: speech, arising from chair, gait, FoG, postural instability, posture.

The mean (SD) right and left stimulation amplitude (mA) across the trials for 180 Hz compared to 60 Hz was 2.91 (0.71) vs. 4.93 (1.19) and 2.63 (0.76) vs. 4.52 (1.27), respectively. Since the differences between hemibodies were significant for most gait parameters (data not shown), the left- and right-side measurements have been analyzed separately.

### Baseline gait characteristics and influence of L-dopa

In the OFF DBS/OFF MED condition, PD patients showed a significant reduction compared to the OFF DBS/ON MED condition in temporal features such as gait speed (left: *F* = 84.85, *p* < 0.0001; right: *F* = 84.15, *p* < 0.0001), single limb support (left: *F* = 23.14, *p* < 0.001), swing time (right: *F* = 27.77, *p* < 0.0001), foot gait cycle duration (left: *F* = 6.93, *p* = 0.009; right: *F* = 5.15, *p* = 0.02), step duration (left: *F* = 5.72, *p* = 0.02), stance (right: *F* = 27.77, *p* < 0.0001, turn speed (*F* = 66.78, *p* < 0.0001), and turn duration (*F* = 22.11, *p* < 0.0001).

Spatial features that were significantly reduced consisted of stride length (left: *F* = 79.77, *p* < 0.0001; right: *F* = 82.16, *p* < 0.0001), foot strike angle (left: *F* = 64.23, *p* < 0.0001; right: *F* = 55.77, *p* < 0.0001), foot elevation (left: *F* = 7.35, *p* = 0.007; right: *F* = 5.88, *p* = 0.02), toe-off angle (left: *F* = 63.1, *p* < 0.0001), lumbar (CRM: *F* = 68.27, *p* < 0.0001; SRM: *F* = 5.17, *p* = 0.02) and trunk (CRM: *F* = 112.83, *p* < 0.0001; SRM: *F* = 30.15, *p* < 0.0001) range of motion, and right arm range of motion (*F* = 57.49, *p* < 0.0001).

All lower limb gait metrics except for cadence and right footstep duration improved with L-dopa. With respect to axial kinematics, lumbar and trunk range of motion and turning dynamics showed a significant improvement with L-dopa compared with the OFF DBS/OFF MED state (Table 2).

**Table 2:** L-Dopa effects on gait kinematics.

		**OFF MED (SE)**	**ON MED (SE)**	***P*-value (OFF MED *v*. ON MED)^a^**
**Spatial features**				
Lower limb	Foot elevation (R), cm	1.24 (0.13)	1.35 (0.13)	0.0156
	Foot elevation (L), cm	1.25 (0.12)	1.35 (0.12)	0.0069
	Toe-off angle (L), deg	26.1 (1.1)	28.4 (1.1)	<0.0001
	Stride (L), m	0.71 (0.04)	0.80 (0.04)	<0.0001
	Stride (R), m	0.69 (0.04)	0.79 (0.04)	<0.0001
	Foot strike angle (L), deg	7.5 (1.4)	10.1 (1.4)	<0.0001
	Foot strike angle (R), deg	6.5 (1.4)	9.6 (1.4)	<0.0001
Lumbar ROM	Lumbar CRM, deg	4.3 (0.4)	5.0 (0.4)	<0.0001
	Lumbar SRM, deg	5.5 (0.4)	5.7 (0.4)	0.0233
	Lumbar TRM, deg	8.0 (0.6)	9.2 (0.6)	<0.0001
Trunk ROM	Trunk CRM, deg	3.8 (0.4)	5.0 (0.4)	<0.0001
	Trunk TRM, deg	7.2 (0.5)	8.3 (0.5)	<0.0001
	Trunk SRM, deg	3.7 (0.2)	4.2 (0.2)	<0.0001
Turns and arm ROM	Turn angle, deg	140.4 (3.1)	144.0 (3.7)	ns
	Arm ROM (R), deg	19.6 (3.0)	26.6 (3.1)	<0.0001
**Temporal features**				
Lower limb	Swing (R), % GCT	35.6 (0.6)	36.4 (0.6)	<0.0001
	Cadence (L), steps/min	95.19 (2.85)	96.38 (2.87)	ns
	Cadence (R), steps/min	95.33 (2.87)	96.22 (2.89)	ns
	Gait cycle duration (L), s	1.33 (0.04)	1.30 (0.04)	0.0087
	Gait cycle duration (R), s	1.33 (0.04)	1.31 (0.04)	0.0237
	Step duration (L), s	0.67 (0.02)	0.65 (0.02)	0.017
	Step duration (R), s	0.66 (0.02)	0.65 (0.02)	ns
	Gait speed (L), m/s	0.58 (0.04)	0.67 (0.04)	<0.0001
	Gait speed (R), m/s	0.57 (0.04)	0.65 (0.04)	<0.0001
	Single limb support (L), % GCT	35.8 (0.60)	36.5 (0.6)	<0.0001
	Stance (R), % GCT	64.4 (0.6)	63.6 (0.6)	<0.0001
Turns	Turn speed, deg/s	101.0 (7.3)	117.5 (7.4)	<0.0001
	Turn duration, s	2.93 (0.10)	2.659 (0.10)	<0.0001

^a^*p* < 0.05.

### Effects of frequency and electrode contact pairs on gait features

**Figure 1:**
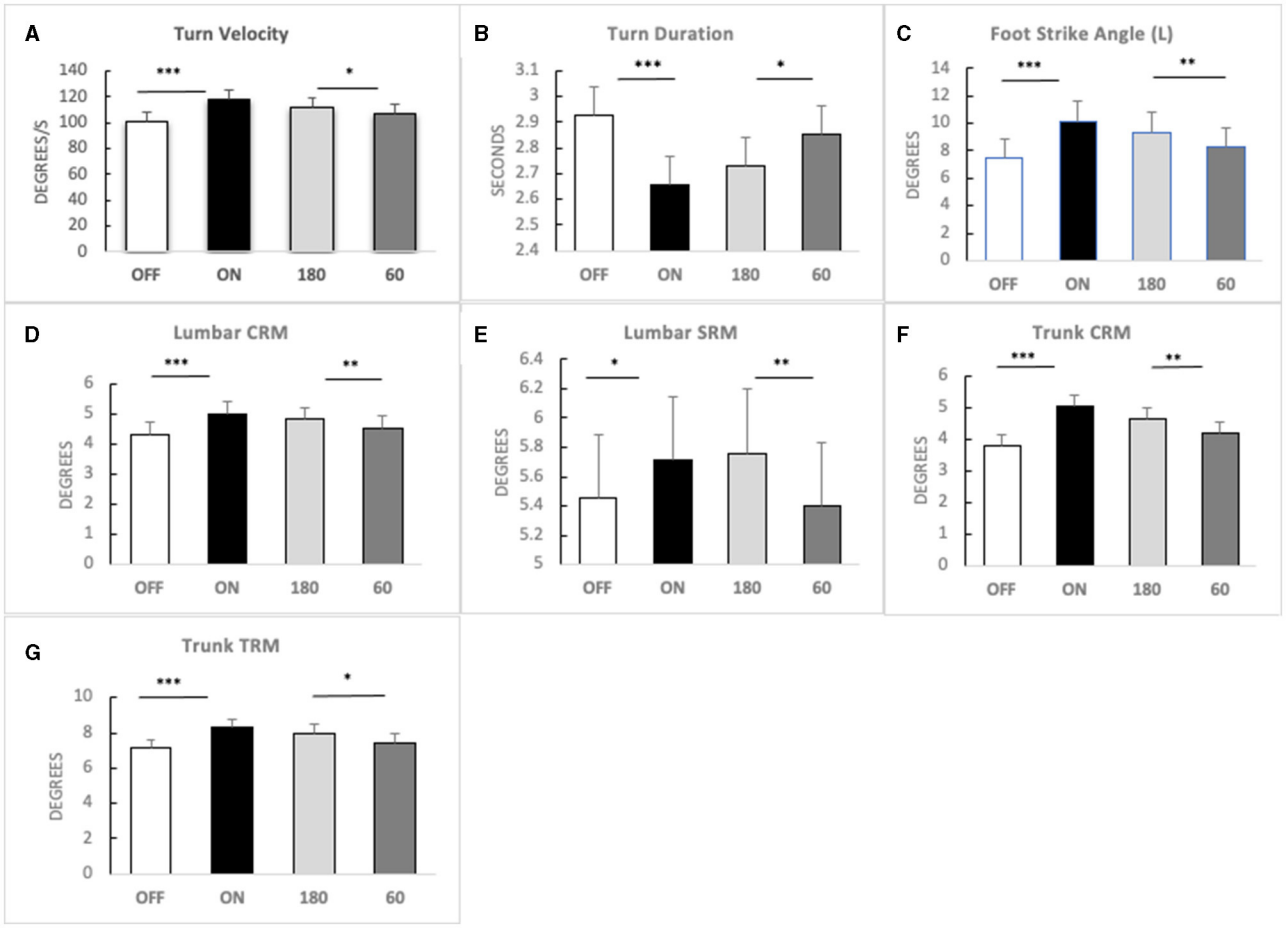
Main effects of high-frequency STN-DBS. Means (bars) and standard errors (whiskers) in four conditions [OFF MED, ON MED, 180 Hz DBS (180), and 60 Hz DBS (60)] for **(A)** turn velocity; **(B)** turn duration; **(C)** foot strike angle—left; **(D)** lumbar coronal range of motion (CRM); **(E)** lumbar sagittal range of motion (SRM); **(F)** trunk coronal range of motion (CRM); **(G)** trunk transverse range of motion (TRM). Significance: LM-ANOVA *p < 0.05, **p < 0.001, ***p < 0.0001.

A three-factorial LM-ANOVA with fixed effects of L-Dopa, frequency stimulation, and contact pairs adjusted for an assistive device revealed a significant effect of stimulation frequency for the following gait features: turn velocity (*F* = 4.40, *p* = 0.0363) and turn duration (*F* = 4.65, *p* = 0.0314); left foot strike angle (*F* = 11.25, *p* = 0.0008); lumbar coronal (*F* = 13.79, *p* = 0.0002), transverse (*F* = 5.71, *p* = 0.0172), and sagittal range of motion (*F* = 10.54, *p* = 0.0012); and trunk coronal (*F* = 14.48, *p* = 0.0002) and transverse range of motion (*F* = 7.46, *p* = 0.0065). High frequency generated a more significant response compared with 60 Hz in these parameters (Figure 1).

**Figure 2:**
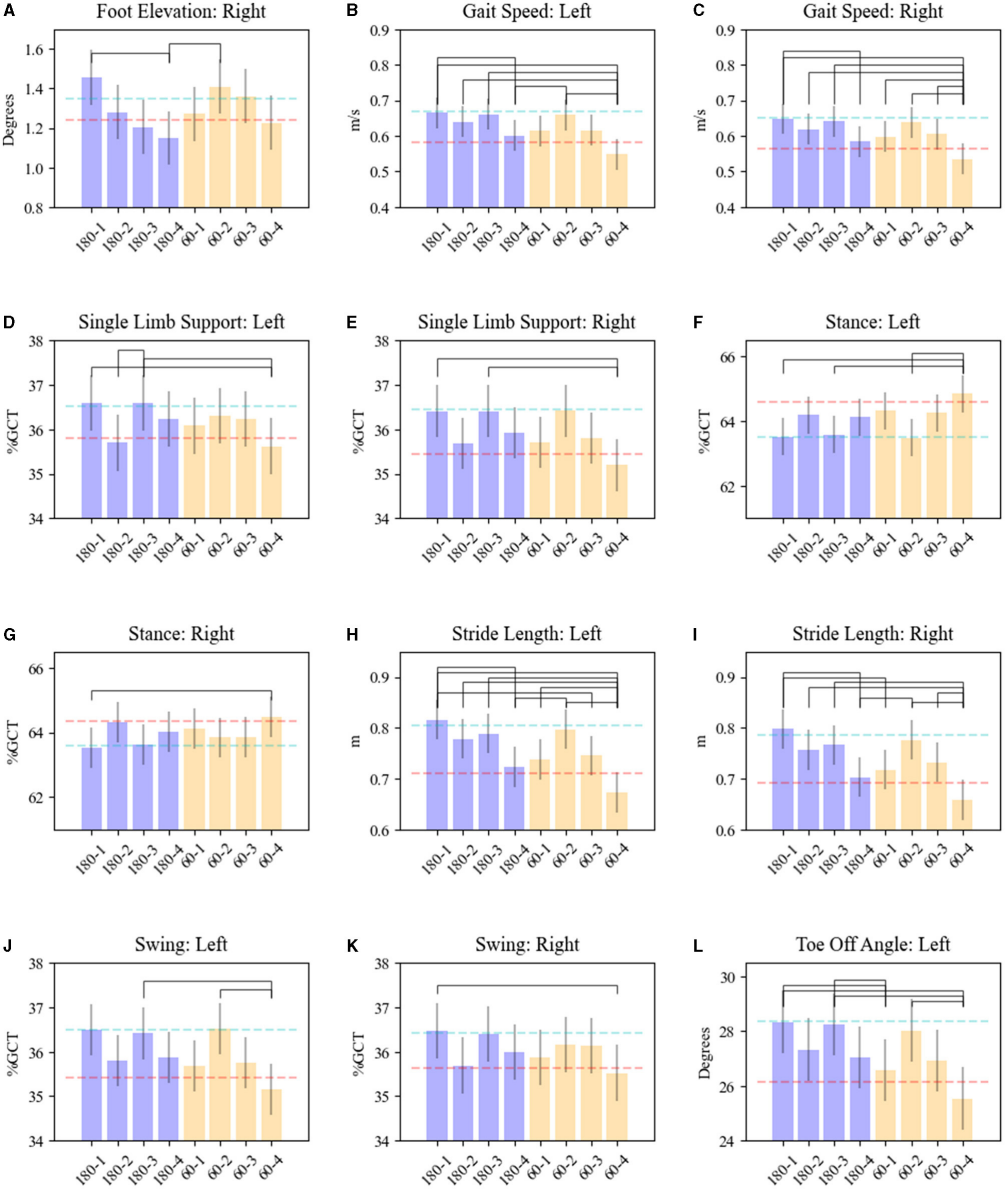
Effects of STN-DBS frequency and electrode contacts on gait kinematics. Results are shown for **(A)** foot elevation—right; **(B, C)** gait speed—left and right foot; **(D, E)** single limb support—left and right foot; **(F, G)** stance—left and right foot; **(H, I)** stride length—left and right foot; **(J, K)** swing—left and right foot; **(L)** toe-off angle—left. OFF MED/OFF DBS mean is shown as the red dash line. ON MED/OFF DBS mean is shown as the blue dashed line. The bars represent means for each parameter related to the combination of HFS (180) or LFS-DBS (60) and electrode contact pairs (1, 2, 3, 4). Whiskers represent the standard error of the mean. Brackets reveal significant pairwise differences between DBS (180/60)-contacts pairs, *p* < 0.05.

Significant interactions between frequency and contact pairs on gait parameters were also observed for foot elevation (right: *F* = 3.31, *p* = 0.02), foot speed (left: *F* = 3.47, *p* = 0.02; right: *F* = 3.21, *p* = 0.02), toe-off angle (left: *F* = 4.37, *p* = 0.005), SLS (left: *F* = 3.81, *p* = 0.01; right: *F* = 5.41, *p* = 0.0011), stance (right: *F* = 2.85, *p* = 0.04; left: *F* = 5.62, *p* = 0.0008), stride length (left: *F* = 4.05, *p* = 0.007; right: *F* = 4.32, *p* = 0.005), foot swing (right: *F* = 2.86, *p* = 0.04; left: *F* = 5.62, *p* = 0.0008), and arm swing range of motion (left: *F* = 3.54, *p* = 0.01). These parameters were significantly worse with both LFS and HFS on dorsal contacts (3 & 4) compared with ventral contacts (1 & 2) (see Figure 2). HFS and LFS at the ventral contact levels showed no comparatively significant response differences among these gait parameters except for left toe-off angle (*p* = 0.02) and right stride length (*p* = 0.0015) by which HFS on contact 1 produced a significant improvement compared with 60 Hz.

Notably, ventral stimulation was associated with improvement in both temporal (foot speed, stance, SLS, and foot swing) as well as spatial (foot elevation, toe-off angle, and stride length) gait parameters compared to dorsal contact stimulation regardless of the stimulation frequency utilized. Dyskinesia was observed in 16% of the SAW trials−83% of which were in the HFS-DBS/ON MED.

### Interaction of frequency and levodopa on gait kinematics

**Figure 3:**
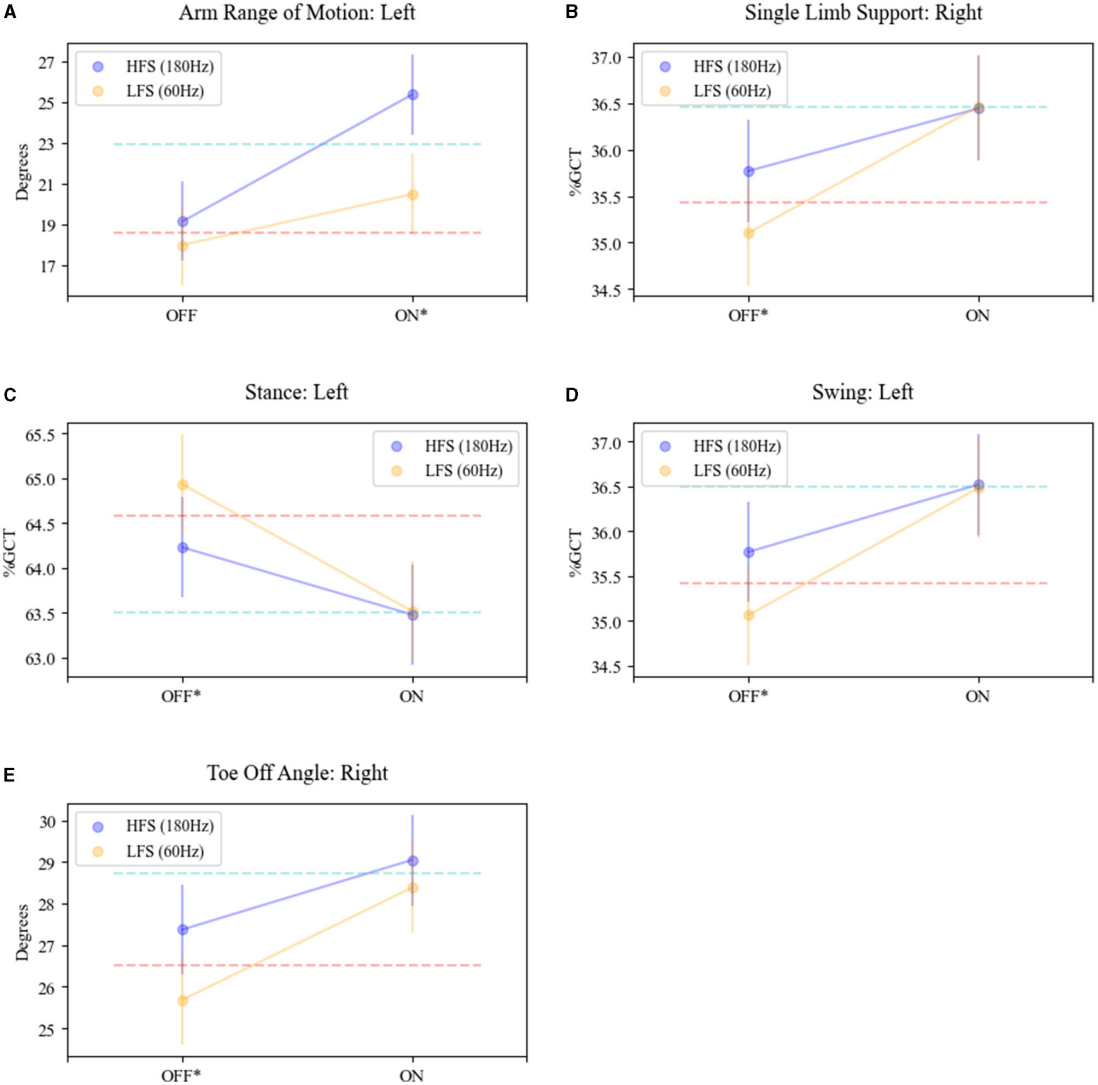
Interaction effect of STN-DBS frequency and L-Dopa on gait parameters. The mean trajectories in the OFF (MED) and ON (MED) for HFS (180; purple plot) and LFS (60, yellow plot) DBS for **(A)** arm range of motion—left arm; **(B)** single limb support—right foot; **(C)** stance—left foot; **(D)** swing—left foot; **(E)** toe-off angle—right foot. OFF MED/OFF DBS mean is shown as the red dash line. ON MED/OFF DBS mean is shown as the blue dashed line. *Significance and interaction effects between conditions OFF MED/ON DBS (180)- OFF MED/ON DBS (60) and ON MED/ON DBS (180)-ON MED/ON DBS (60) determined by LM-ANOVA, *p* < 0.05.

An interaction effect with L-Dopa and stimulation frequency was revealed for toe-off angle (right: *F* = 4.06, *p* = 0.04), SLS (right: *F* = 5.07, *p* = 0.02), foot stance (left: *F* = 4.68, *p* = 0.03), foot swing (left: *F* = 4.68, *p* = 0.03), and arm range of motion (left: *F* = 7.50, *p* = 0.006) (see Figure 3). In detail, the means at 180 Hz frequency in the OFF-MED state were significantly larger for right toe-off angle (adj. *p* < 0.0001), right single limb support (adj. *p* < 0.02), and left foot swing (adj. *p* < 0.0073) and significantly higher for 60 Hz for left foot stance (adj. *p* < 0.0073). There was no significant difference in the means for the left arm range of motion in the OFF MED state. In the ON MED state, the left arm range of motion was the only parameter with a significantly larger mean at 180 Hz compared to 60 Hz (adj. *p* < 0.0001).

This interaction effect demonstrates an additive effect of L-dopa and stimulation. LFS in the OFF MED state was associated with worse mean scores for these specific features but in combination with L-Dopa produced a response that was equivalent to HFS. Many of these features impacted by this synergistic effect were temporal characteristics: SLS-R, stance-L, and swing-L.

## Discussion

In the present study, we analyzed the spatiotemporal effects of L-dopa and STN-DBS in the LFS and HFS states by randomizing stimulation between electrode contact pairs while treating the quantitative gait kinematic data for each hemibody separately. In the OFF DBS/OFF MED state, lower limb kinematics showed a reduction in foot velocity, stride length, SLS, and foot elevation and increased stance percentage of the gait cycle. This kinematic profile mirrors the results of studies highlighting the alterations of gait in PD (Blin et al., 1991; Morris et al., 1998; Hausdorff, 2009). The foot placement dynamics (i.e., foot strike angle and toe-off angle) and lumbar and trunk ROM in the orthogonal planes were also reduced in the untreated condition.

Stride length and gait speed improved with stimulation and L-dopa, while cadence remained unchanged. In contrast to prior studies (Faist et al., 2001; Stolze et al., 2001; Ferrarin et al., 2002; Krystkowiak et al., 2003), these results show that LFS produces a similar response to HFS. Notably, the stimulation benefit was most similar between the two frequency states at the level of the ventral contacts on the electrode, where they approximated the OFF DBS/ON MED response. Cadence regulation with PD has been shown to be no different than in control subjects (Blin et al., 1991). Morris et al. (1998) showed that L-Dopa increased both the intercept and usable range of the stride length–cadence relation toward normal. The inability to modulate stride length amplitude in the hypodopaminergic state arises more from defective scaling (inability to generate appropriate amplitude scaling) (Hausdorff, 2009), which can be restored to a certain degree by low- and high-frequency STN-DBS. We assume that since the subjects were walking at their most comfortable speed during each SAW trial, they had not reached their stride length-cadence breakpoint (Morris et al., 1998). Therefore, it is likely that there was a direct impact of L-dopa and stimulation on stride length, which resulted in increased foot speed.

HFS-DBS also produced significant improvements in arm swing range of motion, trunk and lumbar range of motion, as well as turning kinetics. However, the small differences between the two frequency states across these parameters are of unknown clinical relevance and the presence of dyskinesia, though seemingly mild in severity and observed in 16% of the SAW trials majority in the HFS-DBS/ON MED, is further confounding. Nonetheless, these results may reflect stimulation-related axial amplitude scaling with studies reporting HFS improvement of arm swing, trunk flexion, and rotation and hip ROM (Faist et al., 2001; Ferrarin et al., 2002; Potter-Nerger and Volkmann, 2013; Cossu and Pau, 2017) that may be driving gait velocity improvement as well.

Temporal gait characteristics such as cadence, double limb support, and stride-to-stride variability are typically resistant to L-dopa (Blin et al., 1991; Curtze et al., 2015; Galna et al., 2015) and DBS (Faist et al., 2001; Potter-Nerger and Volkmann, 2013; Cossu and Pau, 2017). However, we show that the combination of L-dopa and STN-DBS (60 and 180 Hz) produced a synergistic effect on several lower limb temporal parameters—SLS-R, stance-L, and foot swing-L—as well as spatial parameters—toe-off angle-R and arm swing ROM-L. The changes in SLS, swing phase, and stance were reported in other studies to be related to the increase in stride length (Elble et al., 1991; Faist et al., 2001). While this may be a plausible rationale, one would have to assume that most of the SAW trials in this study were far from the stride length-cadence breakpoint; however, the lack of cadence changes, the use of supratherapeutic level of L-dopa to achieve the ON MED state, and the unquantified role of a gait training effects, calls this into question. Therefore, we postulate that the additive effects of stimulation and L-Dopa on these temporal parameters offer evidence that such treatment combination may be affecting a similar node(s) within a complex and anatomically distributed locomotor network of gait disorder (Giladi et al., 2013); a network that consists of cortical–subcortical circuits of the sensorimotor cortex and SMA, basal ganglia, and brainstem locomotor areas [e.g., mesencephalic locomotor area (MLR) and pedunculopontine nucleus] (Takakusaki, 2017) critical to maintaining automated gait control. In the dopaminergic depleted state, there is overinhibitory activity of the substantia nigra pars reticulata to the MLR (Sherman et al., 2015) along with over-synchronized beta oscillatory activity within the basal ganglia-thalamo-cortical network associated with motor symptoms in PD (Brown et al., 2001; Weinberger et al., 2006; Ray et al., 2008). Wagner et al. (2022) found that concomitant STN and substantia nigra stimulation was superior to conventional STN-DBS in improving temporal measures of gait in PD patients with freezing episodes and associated this with topographically different beta oscillatory cortical activity.

The parameters with synergistic benefits were found to respond similarly to both HFS and LFS at the ventral contacts without additive benefits from L-Dopa on the contralateral limb. While maintaining equivalent TEED between 60 Hz DBS and 180 Hz DBS, stimulation from ventral contacts had a more positive impact on spatiotemporal parameters compared to stimulation from dorsal contacts. This discrepancy in spatial response to stimulation contradicts the finding of Johnsen (2011) which showed gait improvement associated with dorsal contacts but aligns with the results from Hilliard et al. (2011). The lower limb kinematic responses suggest differential influences of L-Dopa and frequency modulation on each hemibody—underscoring the asymmetric nature of PD gait dysfunction, which has been shown to be a key underlying feature in freezing of gait (Plotnik et al., 2005). Fasano et al. (2011) influenced this asymmetry by reducing stimulation on the side contralateral to the limb with larger stride length and found less stride variability and improved inter-limb coordination as a result.

Furthermore, foot dynamic changes [e.g., heel strike, clearance (elevation), and toe-off angle] demonstrated an ability to distinguish advanced Hoehn and Yahr stages of PD (Schlachetzki et al., 2017) and impact balance and freezing episodes (Pillai et al., 2022). The reversal of foot strike abnormalities among PD subjects on L-dopa has also been reported (Hughes et al., 1990). Our results provide evidence that aspects of foot placement abnormalities—specifically toe-off angle, foot strike, and foot swing—can respond to STN-DBS. Additional investigation on the long-term impact of these results on balance and fall risk mitigation is needed.

The response of temporal gait measures to STN-DBS, especially LFS, is predicated on the dopaminergic environment and the location of the stimulation. Temporal characteristics are postulated to be related to mechanisms underlying balance control (Gabell and Nayak, 1984) as well as the pathogenesis of freezing of gait (Nutt et al., 2011). Even though there was no clear advantage of LFS over HFS in this study, based on the doubling of LFS-DBS amplitude compared to HFS-DBS as well as the role ventral contacts seem to play in the stimulation response, we speculate that LFS (60 Hz)-DBS may be impacting downstream brainstem gait centers differently compared to HFS (180 Hz)-DBS. Blumenfeld et al. (2015) showed that 60 Hz STN-DBS produced a different neurophysiological pattern of beta oscillatory changes in the subcortical network of PD compared to HFS. Additional investigation is needed to explore the long-term implications of LFS compared to HFS STN-DBS on gait kinematics including asymmetry and rhythmicity differences while considering electrode and stimulation location within the gait network.

## Limitations

This study has several limitations. First, the small sample size is of note. However, the within-subject, randomized stimulation approach in the OFF and ON medication conditions enabled us to rigorously assess frequency-based responses along all contacts of the electrodes while controlling for the TEED. The lack of a PD control group along with the fact that each stimulation change lasted several minutes limits the generalizability of the results.

## Conclusion

This study provides evidence of synergism of L-dopa and STN-DBS at both HFS and LFS on lower limb spatial and temporal gait measures—SLS, stance, foot swing, and toe-off angle. HFS-DBS and LFS-DBS produced equivalent effects among all other tested lower limb gait features. Stimulation from ventral contacts of the electrode regardless of frequency condition was associated with more favorable responses compared to dorsal contacts. Lastly, lumbar, trunk, and turning kinematics significantly improved with HFS STN-DBS.

## Data availability statement

The original contributions presented in the study are included in the article, further inquiries including requests for datasets and raw data can be directed to the corresponding author.

## Ethics statement

The studies involving humans were approved by the Feinstein Institutes for Medical Research/Northwell Health and the University of Tennessee at Knoxville. The studies were conducted in accordance with the local legislation and institutional requirements. The participants provided their written informed consent to participate in this study.

## Author contributions

RR: design, execution, analysis, writing, and editing of final version of the manuscript. JW and MK: analysis and editing of final version of the manuscript. TF and MN: execution and editing of final version of manuscript. AK: design, analysis, and editing of final version of the manuscript. All authors contributed to the article and approved the submitted version.
